# cAMP Signaling in Pathobiology of Alcohol Associated Liver Disease

**DOI:** 10.3390/biom10101433

**Published:** 2020-10-11

**Authors:** Mohamed Elnagdy, Shirish Barve, Craig McClain, Leila Gobejishvili

**Affiliations:** 1 Alcohol Research Center, University of Louisville, Louisville, KY 40202, USA; mohamed.elnagdy@louisville.edu (M.E.); shirish.barve@louisville.edu (S.B.); craig.mcclain@louisville.edu (C.M.); 2 Hepatobiology & Toxicology Center, University of Louisville, Louisville, KY 40202, USA; 3 Department of Pharmacology & Toxicology, University of Louisville, Louisville, KY 40202, USA; 4 Department of Medicine, University of Louisville, Louisville, KY 40202, USA; 5 Robley Rex VA Medical Center, Louisville, KY 40292, USA

**Keywords:** G protein coupled receptor (GPCR), adenylyl cyclase (AC), phosphodiesterase (PDE), alcohol-associated liver disease (ALD)

## Abstract

The importance of cyclic adenosine monophosphate (cAMP) in cellular responses to extracellular signals is well established. Many years after discovery, our understanding of the intricacy of cAMP signaling has improved dramatically. Multiple layers of regulation exist to ensure the specificity of cellular cAMP signaling. Hence, disturbances in cAMP homeostasis could arise at multiple levels, from changes in G protein coupled receptors and production of cAMP to the rate of degradation by phosphodiesterases. cAMP signaling plays critical roles in metabolism, inflammation and development of fibrosis in several tissues. Alcohol-associated liver disease (ALD) is a multifactorial condition ranging from a simple steatosis to steatohepatitis and fibrosis and ultimately cirrhosis, which might lead to hepatocellular cancer. To date, there is no FDA-approved therapy for ALD. Hence, identifying the targets for the treatment of ALD is an important undertaking. Several human studies have reported the changes in cAMP homeostasis in relation to alcohol use disorders. cAMP signaling has also been extensively studied in in vitro and in vivo models of ALD. This review focuses on the role of cAMP in the pathobiology of ALD with emphasis on the therapeutic potential of targeting cAMP signaling for the treatment of various stages of ALD.

## 1. Introduction

Alcohol-associated liver disease (ALD) encompasses a broad spectrum of liver diseases ranging from simple steatosis to steatohepatitis to fibrosis and cirrhosis, which could lead to liver failure and hepatocellular carcinoma [1]. Approximately 67.3% of the US adult population consumes alcohol, with 7.4% of the US population meeting the criteria of alcohol abuse or alcoholism [2]. In 2010, the estimated cost of excessive alcohol drinking reached USD 249 billion [3]. Alcohol use remains the most common cause of liver-related mortality in the United States. In Europe, 20–30% of the population consumes excessive amounts of alcohol, with the death toll due to alcohol abuse estimated at 7% in men and 6% in women aged 15–44 years [4]. Long-term heavy drinking causes liver damage via several mechanisms, such as oxidative stress with accumulation of reactive oxygen species (ROS) generated from alcohol metabolism; lipopolysaccharide-induced inflammatory response; acetaldehyde toxicity; and nicotinamide adenine dinucleotide (NAD) depletion, to name a few [5]. ALD could be exacerbated by other coexisting liver conditions such as chronic viral hepatitis and non-alcoholic fatty liver disease (NAFLD) [6]. There is no FDA-approved therapy available for any stage of ALD, and standard of care includes alcohol abstinence and nutritional support as the conventional treatment strategies for ALD in the clinic [5].

Cyclic nucleotides, including cAMP and cGMP, are critical second messengers which regulate numerous intracellular processes and signaling pathways [7]. The role of cAMP in the liver is well documented, including its role in the regulation of metabolic pathways in the liver. Importantly, several studies have been conducted to examine the role of cAMP in ALD.

This review will focus on the relevance of the cAMP-dependent pathways in regulating the pathologic aspects that underlie the development of ALD. Additionally, the review evaluates the potential of targeting cAMP metabolism in the treatment and management of various stages of ALD.

## 2. Alcohol-Associated Liver Disease

ALD remains one of the main causes of liver cirrhosis in the United States and worldwide, and it is the leading cause of liver-related mortality in the USA, especially among younger patients [8]. Unfortunately, accurate assessments of different stages of ALD remain challenging because of the difficulty in identifying patients with early stages of the disease and because of potential social stigma. Long-term drinking causes hepatic inflammation and hepatocyte injury by multiple mechanisms, including accumulation of reactive oxygen species (ROS) causing oxidative stress, and toxicity of both lipopolysaccharide and acetaldehyde [9]. Moreover, pathology is also exacerbated by coexisting conditions such as viral hepatitis, obesity and environmental toxins, among others [6].

### 2.1. Alcohol Metabolism

Alcohol is a polar water- and lipid-soluble substance, which is absorbed through the gastrointestinal (GI) tract and distributed throughout the body. Most (95%) of the alcohol is metabolized by the liver and the remaining 5% is excreted in urine and sweat [10]. Alcohol is metabolized by three enzymes in hepatocytes: alcohol dehydrogenase (ADH), cytochrome P4502E1 (CYP2E1) and catalase. ADH is the cytosolic enzyme which converts alcohol into a highly toxic aldehyde byproduct, acetaldehyde via NAD^+^. Acetaldehyde is oxidized by aldehyde dehydrogenase (ALDH) to acetic acid via another NAD^+^ molecule [11]. Alcohol metabolism is associated with NAD depletion and NADH accumulation, which can affect the activity of some metabolic enzymes relying on NAD^+^. Cytochrome P4502E1 also catalyzes the conversion of alcohol to acetaldehyde. Activity of CYP2E1 increases with chronic alcohol use, whereas it is responsible for only 5% of ethanol metabolism under physiological conditions. The catalase system oxidizes alcohol to acetaldehyde via NADPH [12]. The activity of CYP2E1 and catalase increases only with ethanol concentrations > 10 mol/L, while under normal conditions ethanol is mainly metabolized by the ADH system.

### 2.2. ALD Spectrum

**Figure 1:**
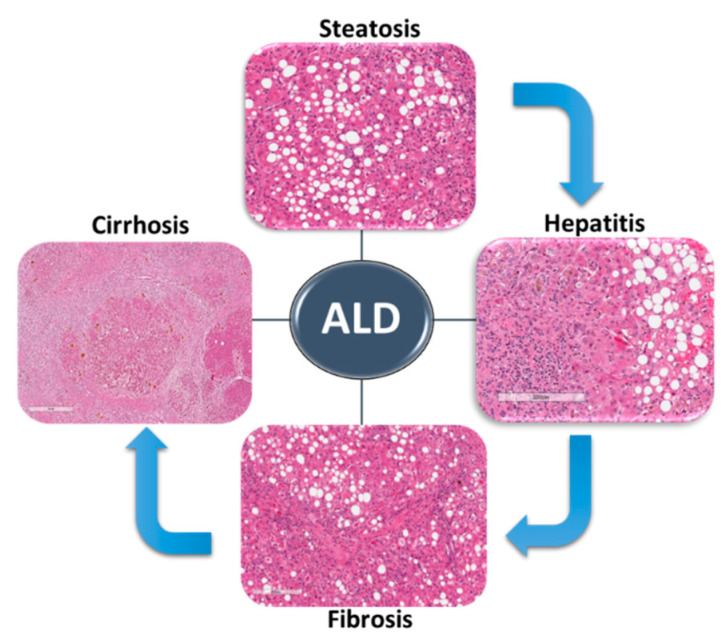
Stages of alcohol-associated liver disease (ALD). Alcohol consumption leads to lipid accumulation in the liver—hepatic steatosis. Alcoholic hepatitis is characterized by immune cell infiltration, hepatocyte necrosis and inflammation of the liver. Fibrosis occurs as a result of advanced, chronic liver injury where collagen and other extracellular matrix proteins (ECM) replace dead hepatocytes. Excessive accumulation of ECM proteins leads to cirrhosis where regenerative nodules are surrounded by fibrous septa. Hematoxylin and eosin-stained liver tissues.

ALD includes a wide spectrum of liver conditions ranging from hepatic steatosis to steatohepatitis and fibrosis, which could progress to liver cirrhosis and even hepatocellular carcinoma (Figure 1) [6]. Alcohol-associated fatty liver (AFL) is the earliest stage of ALD, and it occurs in almost 90% of alcohol consumers [13]. AFL is reversible with alcohol abstinence. Several ethanol-mediated effects contribute to the development of hepatic steatosis in ALD. Increased *de novo* lipogenesis and impaired fatty acid (FA) mitochondrial β oxidation are the main mechanisms of dysregulated lipid metabolism in ALD [14,15,16].

Alcohol-associated steatohepatitis (ASH) is a form of hepatic injury manifesting microscopically as inflammation with ballooning of hepatocytes [17]. Obesity is increasingly recognized as a prominent coexisting risk factor [6,18,19,20]. ASH occurs due to ongoing hepatic inflammation which triggers the release of damage-associated molecular patterns (DAMPs), which lead to stimulation and recruitment of inflammatory cells such as polymorphonuclear leukocytes (PMNLs) and T cells as well as stimulation of Kupffer cells (KCs). This leads to ROS formation, ER stress and accumulation of intracellular cytokeratin inclusion bodies called Mallory–Denk bodies. Factors released by injured hepatocytes and by activated immune cells can activate hepatic stellate cells (HSCs) to initiate the liver fibrosis/cirrhosis cascade [21].

Alcoholic liver fibrosis/cirrhosis is highly associated with heavy alcohol consumption due to accumulation of acetaldehyde produced during alcohol metabolism. Acetaldehyde can destroy the microtubule structure in hepatocytes as well as form acetaldehyde protein adducts which then lead to hepatocyte proteasome inactivation, abnormal DNA repair and mitochondrial damage with impaired oxygen utilization. All these injury mechanisms stimulate HSCs, leading to excessive extracellular matrix protein deposition which, in turn, causes disruption of hepatic architecture with formation of irregular regeneration nodules and bridging portal fibrosis [22].

### 2.3. Mechanisms of ALD

#### 2.3.1. Liver Steatosis

Lipid accumulation or hepatic steatosis is a result of dysregulated lipid metabolism caused by alcohol and its metabolism. Alcohol accelerates hepatic lipogenesis due to increased expression of the lipogenic enzymes, fatty acid synthase (fasn), Acyl CoA carboxylase (Acc), ATP citrate lyase (Acl) and Malic enzyme (Me). Increased expression of lipogenic enzymes is mainly due to induction and activation of a transcription factor, sterol regulatory element-binding protein 1 (SREBP-1, specifically SREBP-1c) [15,23,24]. Moreover, alcohol has also been shown to induce the expression of lipin-1, which plays an important role as an inducer of lipid synthesis in mammalian livers [25]. Alcohol-induced ER stress has been shown to contribute to SREBP-1c induction and is considered to be one of the critical mechanisms in insulin-independent proteolytic activation of SREBP-1c [26]. Alcohol was also shown to inhibit 5’ adenosine monophosphate-activated protein kinase (AMPK) activation, which regulates genes involved in both lipogenesis and beta oxidation [15]. Specifically, AMPK decreases SREBP-1c transcription and ACC activity [27].

Alcohol also decreases hepatic lipolysis by affecting lipophagy, a process of selective autophagy of lipid droplets in hepatocytes by lysosomal lipases. It has been suggested that inhibition occurs due to defective lysosomal formation [28]. Alcohol also decreases the rate of β oxidation by excessive generation of NADH, which inhibits mitochondrial β oxidation enzymes. Alcohol inhibits the transcriptional activity of PPARα with reduced binding to the promoters of the target genes, causing a decrease in the expression of genes regulating FA transport and oxidation [29]. Alcohol consumption also reduces the production of the hormone, adiponectin, which is secreted by adipose tissue to increase FA β oxidation (see the adipose tissue section of this review [30]).

Alcohol causes a decrease in hepatic lipid export in the form of very low-density lipoproteins (VLDL). Triglycerides are packed with proteins by the liver to be secreted to the blood in the form of VLDL where they are metabolized by lipoprotein lipase in the adipose tissue to become intermediate-density lipoproteins (IDL) and low-density lipoproteins (LDL), which are taken up by the liver. Alcohol alters lipid droplet metabolism and decreases VLDL secretion from the liver [31,32]. It was also shown that ethanol exposure increases hepatic uptake of exogenous FA by increased expression of CD36/FA translocase and FA transport proteins which are the main mediators of hepatic FA uptake [33].

#### 2.3.2. Alcohol-Associated Hepatitis and Immune Cells

Kupffer cells (KCs) are the resident macrophages in the liver. Alcohol activates KCs via many mechanisms causing them to release multiple pro-inflammatory cytokines such as TNFα and IL-1β, which lead to the recruitment of inflammatory cells promoting hepatocyte injury.

Chronic ethanol exposure induces alterations in the ultrastructure of enterocytes leading to increased permeability of intestinal mucosa to macromolecules [34,35,36]. Increased gut permeability leads to leakage of bacterial products, e.g., lipopolysaccharide produced by intestinal Gram-negative bacteria, into the liver through the portal blood. Moreover, alcohol was shown to alter the gut microbiota by enhancing the growth of Gram-negative bacteria, a major source of LPS [37]. Induction of inflammatory cytokine production by LPS is mediated through its binding to toll like receptor 4 (TLR4) on the KC surface. This binding is facilitated by other proteins, including LPS-blinding protein, CD14 and myeloid differentiation factor 2 (MD2) [38,39]. Activation of TLR4 leads to a sequence of signaling events causing translocation of the transcription factor, NFκB, to the nucleus, inducing the expression of proinflammatory cytokines including TNFα, IL-1β and IL-6 [40,41].

Additionally, alcohol metabolism via CYP2E1 in KCs leads to the activation of KCs and to the production of TNF and ROS. Alcohol activates inflammasomes, which are intracellular multiprotein complexes activated upon cellular stress, triggering caspase 1 activation and proinflammatory cytokines release [42,43]. In KCs, activation of inflammasomes leads to generation of IL-1β, which causes inflammation by acting on the IL-1 receptor. Serum levels of IL-1β were found to be elevated in ALD patients as well as chronic ethanol-fed rats [44]. Moreover, recombinant IL-1β receptor antagonist markedly attenuated ALD in mice [45]. Kupffer cells undergo polarization to either a pro-inflammatory phenotype (M1) or an anti-inflammatory phenotype (M2) [46,47]. M1-polarized macrophages express pro-inflammatory cytokines such as TNFα, thereby promoting inflammation, tissue injury and inflammatory cellular infiltration. M2-polarized macrophages express anti-inflammatory cytokines such as IL-10, thereby suppressing inflammation and promoting tissue healing [48,49]. Alcohol consumption increases expression of both M1 and M2 polarization in Kupffer cells (hyperpolarization) [50]. However, Kupffer cells from alcohol-fed animals are predominantly M1 phenotype which is associated with hepatic inflammation and tissue injury [47,50,51,52]. In livers of patients who abuse alcohol, increased levels of both M1 and M2 Kupffer cells have been reported [53]. Importantly, it has been shown that M2 Kupffer cells can promote M1 Kupffer cell death via IL-10-mediated apoptosis [52]. Indeed, higher expression of hepatic M2 markers was associated with macrophage apoptosis and less injury in patients who abused alcohol [52]. Shifting the balance from M1 to M2 has been shown to be therapeutic in animal models of ALD [51,54,55].

#### 2.3.3. Fibrosis and Hepatic Stellate Cells

Chronic excessive alcohol consumption can lead to the development of liver fibrosis/cirrhosis. Hepatic stellate cells (HSCs) play the major role in the development of liver fibrosis. They exist as quiescent vitamin A (retinyl ester)-storing cells in the space of Disse, the space between hepatocytes and hepatic sinusoids [56]. Ongoing hepatic injury and inflammation result in uncontrolled proliferation and activation of HSCs, which then leads to liver fibrosis. HSCs undergo a significant phenotypic change into proliferative, contractile and chemotactic myofibroblasts that migrate and accumulate at the site of injury [57]. Myofibroblasts deposit increasing amounts of extracellular matrix proteins (e.g., collagen and fibronectin) which leads to liver scaring associated with significant loss of liver functions [58].

Initiation of HSC activation is largely due to paracrine stimulation; however, the maintenance of activation involves both autocrine and paracrine loops. Paracrine stimuli come from neighboring cells including hepatocytes, endothelial cells, Kupffer cells and platelets: injured hepatocytes release multiple growth factors such as TGFβ; endothelial cells release endothelin and fibronectin [59,60]; KCs also release TGFβ, TNFα and ROS; and platelets release PDGF, TGFβ1 and EGF [61,62].

Acetaldehyde has paracrine effects on HSCs by inducing expression of collagen type 1 genes through a direct transcriptional-dependent mechanism [63,64]. Acetaldehyde also induces TGFβ1 expression which plays a key role in HSCs activation [65]. A study also showed that acetaldehyde primes HSCs to respond to its activating cytokines (e.g., TGFβ1 and PDGF; reviewed in [66]). Interestingly, acetaldehyde was shown to increase TGFβ1-inducible SMAD3 phosphorylation and collagen expression [67]. Moreover, the lipid peroxidation product, malondialdehyde, increased chemokine secretion by HSCs [68]. ROS also were shown to increase the release of profibrotic cytokines (e.g., TNFα and TGFβ1) by Kupffer cells, thereby causing activation of HSCs.

## 3. Cyclic AMP Signaling

**Figure 2:**
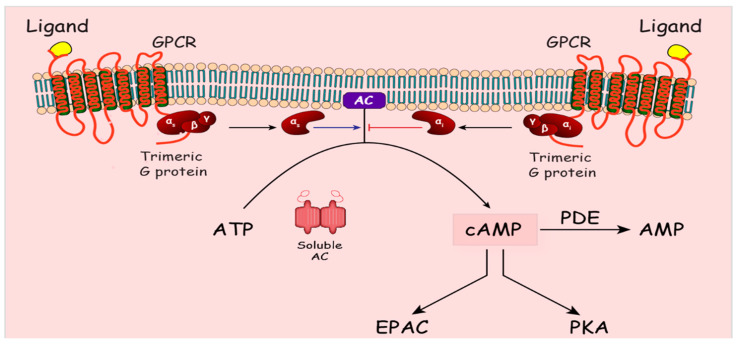
cAMP signaling. Ligand binding to G protein coupled receptor (GPCR) leads to activation of GPCR-bound trimeric G protein and dissociation of α subunit from the βγ dimer. (Gαs) subunit stimulates membrane-bound adenylate cyclase (AC), which converts adenosine triphosphate (ATP) to cyclic adenosine monophosphate (cAMP). (Gα_i_) subunit inhibits AC. Soluble AC, regulated by bicarbonate and calcium, also generates cAMP. cAMP binding to its effectors EPAC (exchange protein activated by cAMP) and PKA (protein kinase A) activate downstream signaling. Phosphodiesterase enzyme (PDE) degrades cAMP to AMP to terminate the signaling.

Cyclic nucleotides are second messenger molecules which relay the signals from hormones and neurotransmitters to the target cells. cAMP was the first second messenger to be identified and described in 1958 [69]. cAMP is generated from ATP by adenylyl cyclase (AC) in response to a variety of signaling molecules. There are nine transmembrane adenylyl cyclases (tmAC) which are differentially expressed and regulated to generate specific responses [70]. In 1975, soluble AC was first described in the cytosol of rat testis [71], but later was also found in the nucleus, mitochondria and centrioles [72]. Soluble AC activity is regulated by intracellular levels of bicarbonate, calcium and ATP [72,73]. Engagement of GPCRs by their specific agonists leads to a conformational change, which activates GPCR-bound trimeric αβγ G protein, where GTP replaces GDP bound to the alpha subunit (Figure 2). This leads the GTP-bound α subunit to dissociate from the βγ dimer. ACs are stimulated mainly by the Gαs dissociated subunit; however, some ACs are also stimulated by the βγ complex [74]. Generated cAMP can stimulate many effector molecules which include protein kinase A (PKA), Guanine nucleotide exchange factor activated by cAMP (EPAC) and cyclic nucleotide gated ion channels. PKA, the most extensively studied effector, is a complex of two regulatory (R) and two catalytic subunits (C). Binding of cAMP to two R subunits causes the C subunits to dissociate [75]. PKA acts on many cytosolic and nuclear substrates. PKA-mediated phosphorylation regulates the activity of numerous metabolic enzymes (e.g., glycogen synthase and phospholipase β2). Regulation of gene expression by PKA is achieved by phosphorylation of cAMP Response Element Binding Protein (CREB), cAMP responsive modulator (CREM) and ATF1. Once phosphorylated, CREB binds to other cofactors, CREB binding protein (CBP) and p300, before binding to cAMP response elements on DNA. The CREM gene acts as a feedback inhibitor for cAMP early repressor protein (ICER) [76,77].

Another important effector for cAMP is EPAC with two genes, EPAC1 and EPAC2, with three transcript variants for each gene [78]. EPAC2 is mainly expressed in liver, brain, pancreas and the adrenal gland, while EPAC1 is expressed ubiquitously. Binding of cAMP to EPAC leads to activation of the Ras GTPases (Rap1 and Rap2) which are also known as cAMP-regulated guanine exchange factors. In addition to their differential cellular expression, subcellular localization of both EPAC1 and EPAC2 determines the specificity of cAMP signaling (reviewed in [78]). They serve as interacting partners for multiple proteins and regulate numerous functions in various organs and systems including the digestive and immune systems [78].

cAMP signaling is fine-tuned by a specific group of enzymes known as phosphodiesterases (PDEs) [79,80,81]. PDEs are a large family of ubiquitously expressed enzymes responsible for termination of cAMP signaling by catalyzing the reaction of cAMP hydrolysis to AMP. There are 11 different PDE families (PDE1 to PDE11) and they differ in their tissue distribution, substrate specificity, subcellular localization and catalytic properties [79,81,82]. They can be grouped according to their substrate specificity: cAMP-specific PDEs including PDE4, PDE7 and PDE8; cGMP-specific PDEs including PDE5, PDE6 and PDE9; and dual specificity PDEs including PDE1, PDE2, PDE3, PDE10 and PDE11. Cells might express several PDE isoforms in various subcellular locations; however, some cells show a relatively abundant expression of a specific PDE (e.g., PDE6 in photoreceptors of the retina). Moreover, expression changes and mutations of multiple PDE enzymes have been linked to several disease states [83].

It is important to point out that generation of cAMP and downstream signaling is specific to the stimulus and cell type. This specificity is insured by the presence of cell-specific GPCRs coupled with Gs proteins and adenylyl cyclases. Some ACs reside in lipid rafts while others are in various compartments of the cell [84]. Additionally, A-kinase anchoring proteins (AKAPs) can interact with ACs to regulate cAMP signaling by creating a scaffold with PKA and its target [84,85]. Importantly, the fine-tuned and specific cAMP signaling is achieved by co-existence of AC with a specific PDE isoform in a scaffold (compartmentalized cAMP signaling), which ensures its spatial, temporal and compartmental downstream signaling activation. A large family of cAMP-specific PDEs provides additional specificity of cAMP signaling. Several studies using PDE4A, B and D knockout mice have shown that these enzymes have non-redundant roles in various cellular and tissue responses [79,86,87,88,89]. Relevant to this review, it has been shown that PDE4B plays an essential role in endotoxin-induced TNF production and toxicity, while PDE4A and D have no effect [87,88].

Several pharmacological approaches have been used to study the role of cAMP signaling in both in vitro and in vivo studies. cAMP analogs, adenylyl cyclase agonists, various PDE inhibitors and GPCR activators are widely used in experimental animal models and are discussed in the following sections. Specific agonists and antagonists of PKA and EPAC have also been developed to study the precise effects of PKA and EPAC in cellular responses. Importantly, several cAMP-elevating agents and isoform-specific PDE inhibitors have been tested and are used clinically to treat inflammation, tissue fibrosis, asthma and neurological disorders (reviewed in [90]). In 2011 and 2014, the FDA approved two orally available PDE4-spesific inhibitors, Roflumilast and Apremilast, to treat severe COPD, psoriasis and psoriatic arthritis. Ibudilast, a dual PDE4/10 inhibitor, has shown anti-inflammatory and neuroprotective properties in humans [91,92,93]. Regarding liver diseases, the broad spectrum PDE inhibitor, Pentoxifylline, has been used in ALD and NASH patients for many years and has shown anti-inflammatory and anti-fibrotic activity [94,95,96,97,98,99].

## 4. cAMP Signaling in ALD

### 4.1. Hepatocytes: Regeneration/Steatosis

The role of cAMP signaling in liver health and disease has been a focus of numerous studies (reviewed in [90]). Due to a critical role of cAMP-dependent signaling in cellular proliferation and differentiation, the potential role of altered cAMP signaling in impaired liver regeneration was examined by Diehl et al. The authors used the chronic Lieber deCarli ethanol feeding model in rats for six weeks followed by 70% partial hepatectomy (PH) [100]. The study reported significantly lower hepatic cytosolic cAMP levels during the first six hours after PH in ethanol-fed rats compared to pair-fed controls, which was later normalized. Underlying the mechanism of diminished cAMP levels was decreased adenylyl cyclase activation due to lower stimulatory G_s_α protein at all time-points after PH, while the expression of inhibitory G_i_2α was increased by 10-fold six hours post-PH in ethanol-fed rats. The authors also noted that ethanol treatment seemed to have a direct effect on AC. Importantly, the results of this study suggested that ethanol-induced desensitization of cAMP signaling may play a role in the impaired liver regenerative response. The critical role of G_s_α in liver regeneration was later shown in two mouse models using hepatocyte-specific G_s_α knockout mice [101]. Specifically, these authors showed that G_s_α inactivation inhibited cyclin-dependent kinase 2, cyclin E and the transition of proliferating hepatocytes from G1 to S phase. The direct effect of ethanol on receptor-stimulated cAMP production in isolated hepatocytes seems to be concentration-dependent [102,103]. While ethanol concentrations up to 50 mM lowered cAMP levels, higher concentrations (50–100 mM) resulted in increased production of cAMP in response to glucagon and adenosine [102,103]. Importantly, ethanol did not affect the basal adenylyl cyclase activity [103].

Oxidative ethanol metabolism by CYP2E1 plays a critical role in alcohol-induced liver injury, as reviewed above. Early studies examining the mechanisms of CYP2E1 turnover showed that cAMP-PKA-mediated phosphorylation of CYP2E1 leads to its proteolysis [104,105]. These observations led to a 1999 study, where investigators used the non-degradable cAMP analog, dibutyryl-cAMP (dbcAMP), to examine the effects of increased cAMP signaling in the development ALD [106]. In this study, intragastric ethanol feeding of rats for eight weeks did not result in significant increases in the liver injury markers ALT and AST despite a significant increase in CYP2E1 expression. However, ethanol-fed rats developed hepatic steatosis and changes in fatty acid composition. Additionally, ethanol feeding decreased ubiquitin expression and ubiquitin conjugates as well as proteasomal enzyme proteolysis. Administration of dbcAMP decreased ethanol-mediated increases in CYP2E1 and CYP4A expression and triglyceride levels. dbcAMP also attenuated the inhibition of the proteasome function and tended to normalize the conversion of 18:2n-6 to 20:4n-6 as well as 18:0 to 18:1 fatty acids, suggesting that cAMP improved delta 5, 6 and 9 desaturases [106]. Investigators also reported that dbcAMP decreased NFkB activation in both pair-fed and ethanol-fed rats. Interestingly, increased cAMP levels/signaling led to increased proliferating cell nuclear antigen (PCNA) antibody staining of both parenchymal and non-parenchymal cells in the liver, including bile ducts. Although the exact mechanisms of this effect were not investigated, the authors speculated that acceleration of the cell cycle and improvement of hepatic blood flow might have contributed to these effects.

The role of GPCRs, specifically adenosine A_1_ and A_2b_ receptors, in the ethanol-mediated increase in hepatic steatosis was later demonstrated in a Lieber deCarli chronic ethanol feeding mouse model [107]. It is important to point out that the A_1_ receptor is coupled to G_i_ proteins, which leads to inhibition of AC and decreased cAMP levels, while A_2A_ and A_2B_ activate G_s_ proteins and increase cAMP/PKA activation. Additionally, both receptors A_1_ and A_2B_ have been shown to modulate MAPK signaling (reviewed in [108]). A study by Peng et al. demonstrated that the human liver expresses all four adenosine receptors (A_1_, A_2A_, A_2B_ and A_3_) [107]; however, steatotic and cirrhotic livers have more A_2A_ and A_2B_ receptors [107]. Using pharmacological and genetic approaches, the paper demonstrated that inhibition of A_1_ and A_2B_ receptors protected mice from developing hepatic steatosis by regulating enzymes involved in fatty acid synthesis and oxidation. In vitro studies using the murine hepatocyte cell line, AML 12, demonstrated that adenosine A_1_ and A_2B_ receptor agonists increased triglyceride accumulation in AML 12 cells. More specifically, adenosine A_1_ agonist promoted fatty acid synthesis via upregulation of SREBP1-dependent increase in lipogenic enzymes, while A_2B_ agonist decreased CPT1A and the AMPK-dependent fatty acid oxidation pathway. However, the effect of ethanol feeding and/or agonists on cellular cAMP levels and signaling in relation to fatty acid metabolism has not been examined.

Given a critical role of cAMP response element binding protein (CREB) in the regulation of fatty acid synthesis and oxidation, the effect of ethanol on hepatocyte pCREB levels was examined in a rat model of ALD using Lieber deCarli ethanol feeding with and without an ethanol binge [109]. Acute ethanol exposure of hepatocytes increased nuclear levels of pCREB, due to activation of p38 MAPK and MSK-1. Interestingly, the data showed that hepatic CREB activation was increased by chronic ethanol feeding but decreased after an ethanol binge in vivo. Importantly, this decrease in nuclear pCREB was associated with a decrease in CPT1A, a rate-limiting enzyme for mitochondrial FA oxidation [110,111]. In this study, the effect of ethanol on hepatocyte and liver cAMP levels was not evaluated. Later, work from our group showed that chronic ethanol feeding significantly decreased cAMP levels in mouse livers as well as in hepatocytes isolated from ethanol-fed mice [111,112]. However, the relative decrease was larger in the whole liver. These results indicated that ethanol might affect other cell types in the liver, which agrees with our previous studies demonstrating that ethanol decreases cAMP levels in Kupffer cells [113]. Importantly, we observed that ethanol decreased hepatic cAMP levels by significantly upregulating cAMP-specific PDE4 both in vivo and in in vitro primary rat and mouse hepatocytes [111,112]. Decreased cAMP levels resulted in lower pCREB levels and decreased CPT1A expression. We confirmed the cAMP/CREB-mediated effect on CPT1A expression in primary mouse hepatocytes by specifically activating and inhibiting PKA/CREB signaling [111]. We then focused our investigation of the role of PDE4-mediated decrease in cAMP signaling on CPT1 dysregulation by ethanol by using pharmacological and gene knockout approaches. CPT1 is regulated by complex transcriptional machinery involving several transcription factors and co-activators (e.g., PPARα, PGC1α, SIRT1, CREB, etc.) [110,114,115]. CPT1A transcriptional activation can be modulated by PKA and EPAC, in various cell types including hepatocytes [110,114,116,117]. In ethanol-induced hepatic steatosis, decreased expression/activity of PPARα and PGC1α has been reported [118,119,120,121,122]. We used a PDE4-specific inhibitor as well as Pde4b knockout mice and showed that the ethanol-mediated decrease in CPT1A expression was prevented via the PPARα/PGC1α/SIRT1 pathway in an in vivo mouse model of chronic Liber deCarli feeding for four weeks [111]. Recently, we recapitulated the same results in a chronic ethanol binge (10 plus one, NIAAA) model [112]. Moreover, a single administration of PDE4 inhibitor prevented the ethanol-mediated decrease in cAMP levels and attenuated liver injury. Our observations showed that PDE4 inhibition was protective due to decreased oxidative and endoplasmic reticulum stress and hepatocyte apoptosis in ethanol-fed mice. Importantly, human livers from alcohol-associated hepatitis patients have much higher expression of PDE4 and decreased cAMP levels [112]. These observations strongly suggest that PDE4-mediated dysregulation of cAMP signaling might play a pathogenic role in human ALD.

Relevant to AFL, recent studies focused on the impaired lipid droplet (LD) lipolysis in hepatocytes as a mechanism of ethanol-mediated hepatic steatosis [123]. It is well established that β-adrenergic stimulation activates adipose triglyceride lipase (ATGL) and hormone-sensitive lipase (HSL) in adipocytes in a PKA-dependent manner, which leads to lipolysis. β-adrenergic stimulation also induces LD lipolysis in hepatocytes; however, hepatocytes exposed to ethanol fail to respond to stimulation to activate PKA and break down LD. Hence, the inability of ethanol-exposed hepatocytes to activate cAMP/PKA in response to β-adrenergic stimulation might also play a role in AFL [123].

The beneficial effect of cAMP signaling via activation of adenylate cyclase (AC) and the PDE3 inhibitor, Cilostazol, on liver injury has also been demonstrated in both in vivo and in vitro ALD models [124,125,126]. Treatment of ethanol-fed rats for the last four weeks of an eight-week study with 14-deoxyandrographolide (14-DAG) attenuated ethanol-induced hepatic apoptosis, oxidative stress and lipid peroxidation via activating AC [126]. Cilostazol decreased ethanol-induced oxidative stress and prevented mitochondrial pathway-mediated apoptosis in hepatocytes [124]. A later study recapitulated the anti-apoptotic effect of Cilostazol in ethanol-treated primary rat hepatocytes [125]. However, the authors suggested that this effect was not mediated by cAMP but rather by AMPK activation by Cilostazol.

### 4.2. Immune Cells/Alcohol Associated Hepatitis (AH)

Research on the role of cAMP signaling in immune cell responses dates to the 1970s, and describes increased antibody formation and anti-tumor activity of immune cells by increased cAMP [127]. One of the first reports of dysregulated cAMP homeostasis in alcohol-associated hepatitis (AH) patients demonstrated decreased cAMP levels in peripheral blood mononuclear cells, suggesting that these patients had an immune dysfunction [128]. Later, it was also shown that lymphocytes from ALD patients had lower basal and adenosine-induced cAMP levels [129]. Bacterial dysbiosis and intestinal barrier damage due to chronic alcohol consumption leads to the leakage of harmful bacteria and bacterial products from the gut into the liver via the portal vein. Exposure of hepatic macrophages, Kupffer cells, to gut-derived bacteria leads to activation of KCs and production of inflammatory cytokines and chemokines. Additionally, ALD-associated peripheral systemic endotoxemia drives immune cells to produce high levels of proinflammatory cytokines (e.g., TNFα, IL-1β, etc.) as discussed earlier in this review. Work from our group showed that chronic ethanol exposure of monocytes and macrophages in culture leads to a significant depletion of basal and endotoxin-inducible intracellular cAMP levels [113,130]. We later showed that this effect of ethanol on cellular cAMP depletion was critically mediated by increased PDE4B activity/expression [130]. Additionally, we showed that Kupffer cells isolated from ethanol-fed rats had much lower levels of cAMP [113]. Importantly, ethanol-exposed monocytes/macrophages produced much higher levels of TNF, which was dependent on PDE4B [130]. Thus, we identified PDE4B as a mediator of ethanol-induced “priming” of monocytes/macrophages. The critical role of cAMP/PDE4 in endotoxin-inducible TNF production has been demonstrated by Conti’s group [87,88]. Specifically, studies done in PDE4A, B and D knockout mice identified PDE4B to be essential in endotoxin-inducible TNF production by both peripheral blood leukocytes and peritoneal macrophages. Significantly, PDE4B knockout mice are protected from endotoxin shock [88]. Later studies by our group examined whether increased cAMP signaling by prostaglandin analog Misoprostol could lead to modulation of inflammation in healthy volunteers. Indeed, administration of Misoprostol significantly reduced LPS-inducible TNF and increased IL-10 production in ex vivo studies. Examination of the mechanism of the misoprostol effect on isolated PBMCs and a murine macrophage cell line demonstrated that the effect of Misoprostol was largely mediated by increased cAMP levels and consequent changes in cyclic AMP response element (CRE) and NFκB activity. Chromatin immunoprecipitation studies confirmed that Misoprostol treatment modulated transcription factor and RNA Polymerase II binding, resulting in changes in TNF and IL-10 mRNA levels [131]. More recently, we evaluated the effect of the FDA-approved PDE4-specific inhibitor, Roflumilast, on LPS-inducible TNFα and IL-1β production in ex vivo studies using whole blood from AH patients [112]. We observed the significant attenuation of both TNF and IL-1β levels by Roflumilast [112]. These studies strongly indicate that PDE4 inhibitors could be beneficial in attenuating inflammation in patients with AH. Regarding anti-inflammatory cAMP signaling in the liver, a recent study showed that AH patients have reduced levels of hepatic beta hydroxybutyrate (BHB), as a result of lower CPT1 expression. BHB is a product of fatty acid β-oxidation with various important signaling properties [132]. Specifically, BHB acts on macrophage hydroxycarboxylic acid receptor 2 (Hcar2) and promotes macrophage polarization to the anti-inflammatory M2 phenotype by a mechanism involving PKA/cAMP [55]. The study showed that mice with reduced BHB levels had an exaggerated hepatic inflammatory response to alcohol, and BHB supplementation reduced alcohol-induced liver injury. As discussed above, cAMP signaling plays an important role in the transcriptional regulation of CPT1A in hepatocytes. Hence, it is possible that increasing cAMP signaling in hepatocytes could also lead to higher hepatic BHB levels.

### 4.3. Stellate Cells/Fibrosis

The anti-fibrotic effect of cAMP effector molecules PKA and EPAC has been demonstrated in various tissue fibroblasts, including HSCs (reviewed in [133,134,135]). Early studies have documented that quiescent HSCs have high levels of pCREB, which decreases upon HSC activation and could be restored with activation of PKA [136,137,138,139]. Our previous studies demonstrated that primary HSCs do not express cAMP-degrading PDE4 when they are quiescent; however, the expression of three PDE4 subfamilies of proteins, PDE4A, B and D, increases upon the early stage of their activation [80]. Importantly, the culturing of freshly isolated rat HSCs in the presence of a PDE4-specific inhibitor significantly attenuated the expression of HSC activation markers, αSMA and Col1a1, and prevented their phenotypic change into myofibroblasts [80]. These data strongly suggest that induction of PDE4 and the consequent decrease in cAMP signaling are required for HSC activation. We also observed a persistent increase in hepatic PDE4 expression in a cholestatic liver injury rat model of fibrosis [80]. We recently examined the expression of hepatic PDE4 in severe AH patient livers with fibrosis and observed a significant upregulation of PDE4 expression (both mRNA and protein) in comparison to donor livers [112]. Several publications have shown the beneficial effects of cAMP-specific PDE inhibitors in attenuating ALD-related hepatic fibrosis in in vivo animal studies [140,141,142].

As we discussed early in this review, regulation of cAMP signaling is fine-tuned by various enzymes in the cells. Recently, Getz et al. suggested that cAMP signaling is also regulated by GIV/Girdin (Guanine nucleotide-binding (G) protein α subunit (Gα)-interacting vesicle-associated protein), the prototypical member of a family of modulators of trimeric GTPases, Guanine nucleotide Exchange Modulators (GEMs) [143]. The same group of investigators demonstrated that GIV mRNA is almost undetectable in normal human livers, but GIV mRNA expression increases with an increasing degree of liver fibrosis [135]. The authors confirmed that hepatic GIV expression correlates with progression of fibrosis in murine models of fibrosis. Importantly, similar to our observations with PDE4 expression, induction of GIV mRNA and protein preceded activation and collagen production in culture-activated HSCs [135]. Induction of GIV enhanced the profibrotic pathways (PI3K-Akt-FoxO1 and TGFβ-SMAD) and inhibited the antifibrotic (cAMP-PKA-pCREB) pathways via activating inhibitory G_i_ in HSCs.

cAMP/EPAC signaling as a regulator of fibrosis in different tissues is also well recognized [133,134]. Mechanisms of EPAC-mediated regulation of fibrosis include activation/differentiation of tissue resident cells, epithelial–mesenchymal transformation (EMT) and recruitment of bone marrow progenitors [133,134]. TGFβ1, the most potent profibrogenic cytokine, decreased EPAC1 expression in fibroblasts [134,144]. A critical mechanism of EPAC-mediated effects on fibroblast activation seems to be signaling of a small GTPase, Rho-A kinase (ROCK) [144]. Early studies have identified Rho-kinase in HSCs as a regulator of actin cytoskeleton reorganization leading to phenotypic change of HSCs to become myofibroblasts [145]. It has also been shown that fibrotic livers from both humans and rodents have decreased levels of EPAC which correlate with increased levels of phospho-Myosin Light Chain (MLC), a downstream target of ROCK1 [144]. Later studies confirmed that Rho-kinase signaling regulates HSC activation and migration [146,147,148]. Several other studies demonstrated the beneficial effects of selective delivery of Rho kinase inhibitor to HSCs on the development of hepatic fibrosis in vivo [146,149,150,151].

The role of GPCRs and cAMP signaling has also been investigated in hepatic fibrosis. Specifically, the role of adenosine receptors has been extensively studied (reviewed in [152]). Adenosine levels increase upon hepatocyte injury, and this can activate adenosine receptors present on HSCs [107,152]. As discussed above, of four adenosine receptors, human steatotic and cirrhotic livers have increased numbers of A_2A_ and A_2B_ receptors [107]. The A_2A_ receptor, which is coupled to stimulatory Gs protein and increases cAMP levels, is a high-affinity receptor activated by nanomolar ligand concentrations, while the A_2B_ receptor is a low-affinity receptor which requires micromolar concentrations of ligand [153,154]. Several studies have found that activation of the A_2A_ receptor promotes HSC activation and fibrosis [155,156,157,158,159]. However, other studies showed that adenosine or its derivatives have opposite effects on fibrosis [160,161,162]. Additionally, adenosine-induced cAMP/PKA signaling has been shown to desensitize endothelin A receptor in activated HSCs leading to decreased contractility [163].

There are conflicting reports on the role of adenosine receptors in HSC activation in response to the alcohol metabolite acetaldehyde. Caffeine, which has been shown to be beneficial in liver fibrosis (reviewed in [164,165,166]), has been reported to block A_1_, A_2A_ and A_2B_ receptors [167]. However, coffee has also been demonstrated to inhibit PDE activity and increase cAMP levels in several studies [168,169,170,171]. Hence, some of the studies using caffeine to antagonize adenosine receptors could be difficult to interpret. A study from 2014 showed that caffeine and an A_2A_ receptor antagonist inhibited the effect of acetaldehyde-induced cell proliferation and procollagen type I/III expression in the rat stellate cell line HSC-T6 by decreasing cAMP levels [156]. The authors also found that caffeine and the A_2A_ antagonist decreased A_2A_R mRNA expression in acetaldehyde-treated cells. The same group later showed that acetaldehyde significantly increased A_1_ and A_2A_ in primary rat stellate cells without any changes in A_2B_ and A_3B_ [155]. Surprisingly, acetaldehyde-induced activation of HSCs was attenuated by using antagonists for both receptors despite their opposite effects on cAMP signaling. The authors speculated that the effect of the A_1_ receptor antagonist on acetaldehyde-induced HSC activation may be cAMP-independent. In line with the role of EPAC in fibrogenesis, acetaldehyde had opposing effects on two different isoforms of EPAC: EPAC1 was decreased, while EPAC2 protein was elevated in primary rat HSCs treated with acetaldehyde [172]. Stimulation of the EPAC1/Rap1 pathway decreased proliferation of HSCs, αSMA expression and collagen type I and III synthesis after exposure to acetaldehyde [172]. Direct transcriptional upregulation of collagen genes by acetaldehyde has also been described in HSCs [64,173]. Specifically, acetaldehyde-responsive element (AcRE) has been identified in promoter regions of *COL1A1* and *COL1A2* genes [64,173]. These regions overlap with other critical transcription factor binding sites for TGF-β1 and TNF-α [64,173,174]. Importantly, these studies suggested that acetaldehyde initiates the fibrogenic process in HSCs and primes them to respond to TGF-β1. Therefore, the acetaldehyde effect on HSCs could be independent of adenosine receptor activation. 

We summarized findings of the effects of alcohol on cAMP signaling in various cell types and processes in Table 1.

## 5. Conclusions

Our understanding of the regulation of cAMP signaling has greatly improved over several decades. Hence, it is important to comprehensively evaluate diverse findings from a new perspective, which is based on current knowledge of the very complex regulatory mechanisms of the compartmentalized signaling of cAMP. Several human and animal studies have shown that alcohol affects cAMP metabolism by changing the expression/activity of GPCRs, ACs and PDEs. It is clear that cAMP signaling is critical in modulating major pathogenic pathways in ALD such as inflammation, steatosis, fibrosis and even liver regeneration. cAMP-elevating agents invariably result in decreased proinflammatory response in monocytes and Kupffer cells. Beneficial effects of cAMP signaling on lipid metabolism and fibrosis are also evident. However, there are conflicting results related to the role of adenosine receptors in ALD. It is important to point out that adenosine receptors are expressed on many cell types and have distinct effects in different cells (reviewed in [175]); hence, the use of cell-specific knockouts will be useful in better understanding their role. Moreover, repeated ligand exposure could lead to desensitization of adenosine receptors and cause tolerance [175]. The challenges in interpreting extracellular adenosine signaling in liver fibrosis have recently been reviewed [152].

Several studies have demonstrated the beneficial role of PDE inhibitors in animal models of ALD. A broad spectrum PDE inhibitor, Pentoxifylline, has shown some benefit in ALD patients. There are several specific PDE inhibitors either already approved by the FDA (Cilostazol, Roflumilast, Apremilast) or used in clinical trials (e.g., Ibudilast). However, there are no human trials testing the potential benefits of these inhibitors in ALD patients. Our group identified the pathogenic role of PDE4 in the development of different stages of ALD using both pharmacological and genetic approaches. However, future studies using cell-specific gene knockouts will be useful to identify the role of PDE4 in a multifactorial disease such as ALD. Due to the ubiquitous nature of the expression of PDE4 enzymes, their role in alcohol effects on adipose tissue and the gastrointestinal tract should be carefully evaluated. Additionally, more recent studies have identified non-coding RNAs which play significant roles in several pathogenic pathways in ALD. The effect of cAMP on the regulation of these non-coding RNAs is starting to be examined [176].

In summary, ALD is a major worldwide health problem with no FDA-approved therapy. Therefore, there is an urgent need to identify therapeutic targets. We suggest that modulation of cAMP signaling has the strong potential to be an effective therapeutic strategy for the treatment of ALD.

## Figures and Tables

**Table 1:** Effect of ethanol on cAMP signaling in ALD.

**Hepatocytes**	
**Liver regeneration**	Chronic alcohol ↑ Gαi and ↓ Gαs ➔ ↓ cAMP ➔ ↓ CDK2 and cyclin E ➔ ↓ Cell cycle G1 to S transition ➔ impaired liver regeneration
**Ethanol metabolism**	dbcAMP decreased ethanol-mediated increase in CYP2E1
**Cell Proliferation**	dbcAMP increased PCNA antibody staining in parenchymal and non-parenchymal liver cells ➔ ↑ Cell proliferation and regeneration
**Fatty acid synthesis**	A1 agonist ➔ (? ↓ AC, cAMP) ➔ ↑ SREBP1 ➔ ↑ Fatty acid synthesis
**Fatty acid oxidation**	- A2B agonist ➔(? ↑ AC, cAMP) ➔ ↓ CPT1A and AMPK ➔ ↓ Fatty acid oxidation - ↑ PDE4B ➔ ↓ cAMP ➔ ↓ PKA/CREB phosphorylation ➔ ↓ CPT1A
**Lipid droplet lipolysis**	Ethanol decreased β-adrenergic-inducible PKA activation and lipid droplet lipolysis
**Immune cells**	
**Immune cells response**	- Chronic ethanol ➔ ↑ PDE4 ➔ ↓ cAMP in monocytes and macrophages/Kupffer cells - ↑ PDE4 ➔ ↓ cAMP ➔ ↑ TNF production in response to endotoxin - Roflumilast (PDE4 inhibitor) ➔ ↓ IL1β and TNF in AH patient blood ex vivo
**Macrophage M1 to M2 polarization**	↓ Beta Hydroxybutyrate (BHB) ➔ ↓ Hcar2/cAMP-mediated M1 to M2 polarization
**Stellate cells**	
**HSC activation**	- ↓ pCREB level ↑ PDE4 ↑ GIV/Girdin ↑ Gi
**Acetaldehyde-induced HSC activation**	- ↑ A2A receptor activation ➔ ↑ HSCs/fibrosis - Adenosine ➔ ↓ liver fibrosis - Adenosine ➔ ↑ cAMP/PKA ➔ ↓ ET-A receptor ➔ ↓ HSC contractility - Caffeine ➔ ↓ A1, A2a, A2b ➔ ↓ Liver fibrosis and acetaldehyde-induced HSC activation
**HSC proliferation and motility**	- Acetaldehyde ➔ ↓ EPAC1 and ↑ EPAC2 ➔ ↑ HSC proliferation, ↑ αSMA, ↑ Collagen

This table summarizes findings of the studies examining the effects of cAMP signaling on various cell types and processes in ALD. ↑ indicates increase; ↓ indicates decrease; ➔ indicates the process leads to a specific effect; ? indicates that expected effects of the agonists on adenylate cyclase (AC) and cAMP levels were not evaluated.
